# Tungsten Distribution in Soil and Rice in the Vicinity of the World's Largest and Longest-Operating Tungsten Mine in China

**DOI:** 10.1371/journal.pone.0091981

**Published:** 2014-03-18

**Authors:** Chunye Lin, Ruiping Li, Hongguang Cheng, Jing Wang, Xiao Shao

**Affiliations:** 1 State Key Joint Laboratory of Environmental Simulation and Pollution Control, School of Environment, Beijing Normal University, Beijing, China; 2 Key Laboratory of Land Use, China Land Surveying and Planning Institute, Ministry of land and Resources, Beijing, China; Institute of Genetics and Developmental Biology, Chinese Academy of Sciences, China

## Abstract

The objective of this study is to investigate tungsten (W) contamination in soil and its enrichment in rice in the area of the world's largest and longest-operating W mines in China. Root zone soil and rice plants were sampled at 15 sites in the agricultural field adjacent to W mines and analyzed for Al, Fe, Mn, Sc, and W contents and W chemical forms in the soil samples and W contents in the rice root, stem, leaf, and grain samples. Results showed that W content in the soil ranged from 3.99 to 43.7 mg kg^−1^, with more than 90% of W in the residual fraction, showing its low mobility and bioavailability. Average W contents in the rice root, stem, leaf, and grain were 7.06, 2.34, 4.76, 0.17 mg kg^−1^, respectively. In addition, they were linearly independent of W content and chemical forms in the soil. Average enrichment factor values were 0.39, 0.13, 0.28, and 0.01 for the root, stem, leaf, and grain, respectively. In can be concluded that W mining activity in the Dayu county contaminated the nearby agricultural soil and led to W bioaccumulation in the rice. This may pose a health risk to residents via food and soil ingestion, which should be a focus of scrutiny.

## Introduction

In recent years, the biogeochemistry of tungsten (W) has become a matter of increasing concern due to the scrutiny of a children leukemia cluster in Nevada, its toxicity to organisms, and ubiquitous presence of this element in the environment as a result of geogenic and anthropogenic processes [1]–[5]. Whereas accumulation of some heavy metals in soils and organisms has been well studied [6]–[11], the researches on W accumulation in soils and plants are extremely limited.

In general, the content of W in non-polluted soils is low, ranging from 0.4 to 5.0 mg kg^−1^ [6]. However, a few previous studies showed that the concentration of W in the soils around the W mining and/or smelting sites was elevated due to the emission of W during the mining and/or smelting processes [12]–[18]. For instance, high average W concentration (56 mg kg^−1^) was reported for the topsoils in the vicinity of mining/smelting sites in North Queensland, Australia [14]. An investigation detected similar levels (10–67 mg kg^−1^) of W content in top soils collected from four random locations in Fallon, Nevada within a few miles from a W refining plant [3]. Up to 150 mg kg^−1^ of W in the soil at an industrial production site for W trioxide in Switzerland was detected compared to geogenic background concentrations of 1 to 2.5 mg kg^−1^ [19].

Mobility and bioavailability of inorganic contaminants in soils depend on their chemical forms. However, studies on the chemical forms and bioavailability of W in soils are extremely limited. Wilson and Pyatt [15], [17] investigated chemical forms and bioavailability in the soils adjacent to W mines, showing that the bioavailability and mobility of W was much lower in the acidic soils than in alkaline soils. Soils surrounding a W ore-processing plant contained total W in the range of 100 to 200 mg kg^−1^, of which 30% is water soluble, 15% bound to Fe oxides, and 5% fixed by organic matter [6].

W uptake by plants, especially by agricultural crops, is of concern because of the potential for W to enter the food supply [20]. Land plants growing in the uncontaminated soils by W generally contain low W, being less than 0.1 mg kg^−1^ [16], [19], [21]–[23]. High concentration of W in the contaminated soils generally elevated the concentration of W in the wild land plants such as trees, shrubs, and grasses [12]–[18]. For example, *Calluna Vulgaris* growing in the soil of Carrock Fell Mine, UK containing 1169 mg kg^−1^ of W had 655, 48.9, and 124 mg kg^−1^ of W in its root, woody part, and leaves, respectively [15]. In addition, the concentration of W in the plants growing in the tungstate or metallic W added soils increased with the concentration of W in the soils [2], [20], [24]–[30]. For example, bioaccumulation of W into *Brassica Oleracae* leaves was dose-dependent with soil W concentration, ranging from 278 to 1420 mg kg^−1^ of W [30].

The literature findings discussed above are confusing as to W chemical forms in the soils. No one determined W concentration, distribution, and enrichment in rice, one of major food around the world.

China is the world's largest W producer and consumer. World tungsten supply is dominated by Chinese production and exports. In 2010, world W mine production was 61,000 t, of which 52,000 t is from China. Ganzhou in the south of Jiangxi province, being the birth place of Chinese W industry, is extremely rich in W sources. W output in Ganzhou has been at the top in China since W was discovered in Xihuashan mine in 1907 in the Dayu county, Ganzhou.

The objectives of this study were to investigate W content and distribution in the agricultural soils and rice in the vicinity of the largest W mine in China, with the longest W mining history. In addition, relationships between W contents in rice and soils were developed. This information is important to quantify W exposure to habitants via food chain.

## Materials and Methods

### Study site

**Figure 1:**
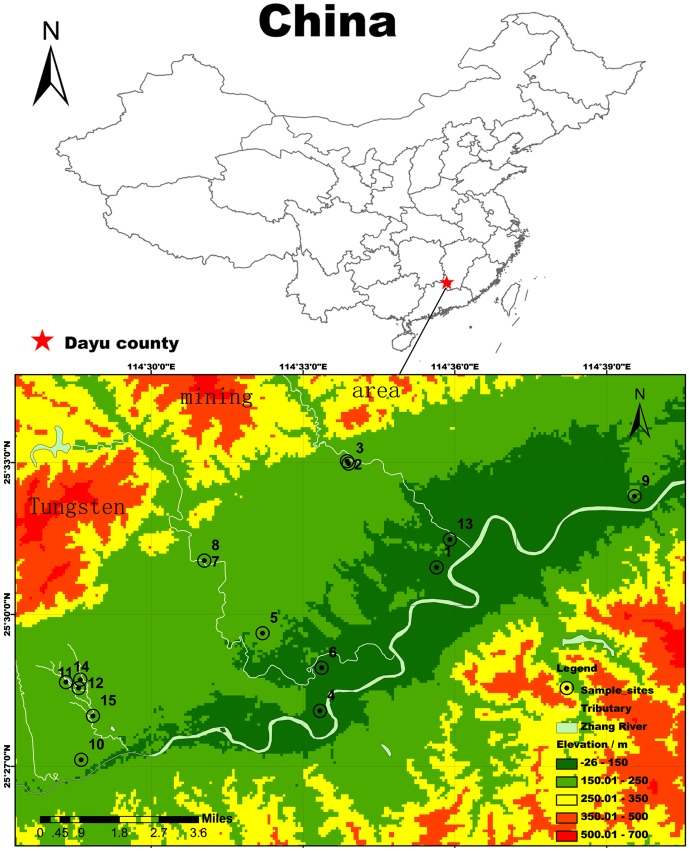
Schematic graph of W mine locations and soil sampling areas.

The study site is situated in Dayu county, the southern Jiangxi province of southern China. It is characterized by subtropical monsoon climate, with average annual precipitation and temperature of 1591.5 mm and 18.5°C, respectively. Major crops are paddy rice and maize. There are three major tungsten mines in Dayu county: Xihuashan, Dangping, and Piaotang, among which Xihuashan is the first tungsten mine operated in China (Fig. 1.). Tungsten occurs in wolframite (Fe,MnWO_4_) in these mines. Local farmers usually irrigate cultivated field with river water originated from the W mining area. No specific permissions were required for the studied area (25^o^27′08′′−25^o^33′02′′N, 114^o^28′19′′−114^o^39′33′′E). This field study did not involve endangered or protected species.

The soil at Dayu county generally contains soil organic matter of 2.76% to 6.26% and is characterized with acidic pH in the range of 5.09 to 6.28 and sandy loam texture [31].

### Soil and plant sampling and analysis

Root zone soil and the rice plants were sampled at 15 sites in the agricultural fields adjacent to W mines. In order to investigate the transfer of W from soils to rice plant, we think it better to collect root zone soil samples. When rice is ready for harvest, rice plants were pulled out together with their roots and root-attached soils. The plants were vigorously shaken by hand and then washed with a little deionized water to remove the root-attached soil. The removed soil and rice plants were put into the plastic bags separately and brought to lab. The soil samples were air-dried in lab, crushed, passed through 0.149 mm sieve (100 mesh), and stored in glass bottles. The rice plants were washed in deionized water and oven-dried at 70°C. Then, root, stem, leaf, and grain (without husk) of the rice plants were separated, ground, and stored in glass bottles.

The pH value of each soil sample was analyzed in a 1∶10 solid/liquid ratio suspension (left for ∼0.5 h) using a combination pH electrode (Orion, USA). Portions of the soil samples were digested with HNO_3_–HF–HClO_4_ [32]. The Al, Fe, and Mn in the extracts were measured with ICP-AES (IRIS Intrepid II, Thermo Electron), while W and Sc were measured with ICP-MS (X Series II, Thermo Electron). The soil mineral matrix elements (Al, Fe, Mn, and Sc) were determined in order to show the basic geochemical compositions of the soils. Together with digestion and measurement of our soil samples, one reference soil (GSD7a) (triplicate), provided by Institute of Geophysical and Geochemical Exploration, Chinese Academy of Geological Sciences, was digested and analyzed to check the analytical quality. Average relative errors were 3.6 (1.9′5.8)%, 4.0(3.5∼4.5)%, 3.9(3.2∼4.7)%, 6.5(4.1∼8.2)%, and −1.1(−6.2∼2.0)% for Al, Fe, Mn, Sc, and W, respectively.

Portions of the root, stem, leaf, and grain samples were digested with concentrated HNO_3_ and H_2_O_2_, and then concentrations of W in the extracts were measured with ICP-MS (X Series II, Thermo Electron).

In order to evaluate mobility and potential bioavailability of W, chemical forms of W in the soil samples were analyzed using a selectively sequential dissolution (SSD) method developed by Tessier et al. [33]. The chemical forms were operationally fractioned into the five phases: (1) soluble and exchangeable fraction (EXC), (2) bound to carbonate minerals (CARB), (3) bound to oxides (OX) (4) bound to organic matter (OM), and (5) residual fraction (RES). The selective sequential extractions were conducted in centrifuge tubes (polypropylene, 50 mL). After each successive extraction, separation was performed by centrifuging at 10,000 rpm for 10 min using a Xiang Yi centrifuge at room temperature. The supernatants were then removed with a pipette and filtered through a 0.45-μm membrane. The W concentration in each solution was determined using ICP-MS (X Series II, Thermo Electron). In this study, only the first four forms were measured and the contents of W in the residual fraction were calculated by total content minus sum of the first four forms.

## Results and Discussion

### Contents of mineral matrix elements and tungsten in the soil

Table 1 summarizes levels of pH, some mineral matrix elements and W in the soils collected in the agricultural fields near the W mines. Soil pH value ranged from 4.95 to 5.92, showing its acidic property. The mineral matrix elements Mn, Sc, Al, and Fe contents in the soil ranged from 68.8 to 718.8 mg kg^−1^, 5.87 to 14.21 mg kg^−1^, 2.47 to 7.69%, and 0.93 to 5.04%, respectively. The mean and median contents of Mn, Sc, Al, and Fe were generally lower than their background contents in the soils of the Jiangxi province, China, and world (except Sc) [34], [35]. However, mean and median contents of W in the soil were 16.57 and 15.20 mg kg^−1^, respectively; much higher than its background contents in the soils of the Jiangxi province, China, and world [34], [35]. Therefore, the agricultural soil adjacent to W mines in Dayu county is contaminated by W.

**Table 1:** Contents of mineral matrix elements and W in the soil.

Sample	pH	Mn	Sc	Al	Fe	W
		mg/kg	mg/kg	mg/kg	mg/kg	mg/kg
**1**	**5.60**	718.60	14.21	7.69	5.04	8.21
**2**	**5.57**	181.70	8.51	4.17	1.30	43.65
**3**	**5.26**	161.80	6.42	2.72	1.73	16.22
**4**	**5.64**	435.10	9.96	5.52	2.26	28.00
**5**	**5.07**	168.60	5.96	2.78	1.77	12.59
**6**	**5.42**	316.50	10.19	4.84	2.69	17.04
**7**	**5.45**	145.40	5.91	2.47	0.93	15.20
**8**	**5.38**	158.50	6.49	2.90	1.02	15.48
**9**	**5.36**	405.30	10.11	5.79	2.73	37.95
**10**	**5.92**	179.40	8.74	4.19	1.68	10.41
**11**	**5.37**	68.76	5.87	2.60	1.31	5.27
**12**	**5.51**	77.79	6.96	3.32	1.21	3.99
**13**	**4.95**	180.40	8.42	4.29	2.40	4.26
**14**	**5.00**	82.84	6.06	2.76	1.33	4.98
**16**	**5.24**	170.00	8.92	3.96	1.88	25.36
**Mean**	**5.38**	**230.05**	**8.18**	**4.00**	**1.95**	**16.57**
**Median**	**5.38**	**170.00**	**8.42**	**3.96**	**1.73**	**15.20**
**Max**	**5.92**	**718.60**	**14.21**	**7.69**	**5.04**	**43.65**
**Min**	**4.95**	**68.76**	**5.87**	**2.47**	**0.93**	**3.99**
**Jiangxi^1^**		**328.00**	**10.31**	**8.60**	**2.88**	**5.28**
**China^1^**		**583.00**	**11.10**	**6.62**	**2.94**	**2.48**
**World^2^**		**1000.00**	**7.00**	**7.10**	**4.00**	**1.50**

1: Data are cited from reference [34].

2: Data are cited from reference [35].

In general, soils in the areas of W mining and/or smelting contained high W, e.g., 24.7–78.4 mg kg^−1^ for Mt Carbine Mine (Queensland, Australia) [14], 10 to 67 mg kg^−1^ for Fallon (Nevada, USA) [3], 1593 mg kg^−1^ for Carrock Fell Mine (Cumbria, UK), 920 mg kg^−1^ for Trumbull Old Mine (Connecticut, USA), 116 mg kg^−1^ for Devon Great Consols (Tmar, UK) [16], and 150 mg kg^−1^ for an industrial production site for W trioxide in Switzerland [19]. In the fallowland soil down the hill from the Höllberg W mine in Germany, W content ranged 15.9 to 101.4 mg kg^−1^ [18]. W content ranged from 3.99 to 43.65 mg kg^−1^ in the agricultural top soils adjacent to W mines in Dayu county, similar to the above mentioned contents.

### Chemical forms of W in the soils

Generally, W in the soils was mostly bound in the RES fraction (89 to 97% of total W), while 2.9 to 10.3% of total W was associated with soil organic matter (Table 2.). Tungsten associated with EXC, CARB, and OX fraction was generally less than 0.5% of total W.

**Table 2:** Proportion of W in each chemical forms in the soil.

Sample	EXC	CARB	OX	OM	RES
	%	%	%	%	%
**1**	0.02	0.02	0.08	2.87	97.01
**2**	0.13	0.03	0.43	7.62	91.80
**3**	0.02	0.01	0.23	6.16	93.58
**4**	0.02	0.01	0.38	6.27	93.33
**5**	0.02	0.01	0.20	6.31	93.46
**6**	0.03	0.01	0.21	5.14	94.60
**7**	0.11	0.02	0.36	7.62	91.88
**8**	0.10	0.02	0.37	10.25	89.26
**9**	0.03	0.01	0.35	5.51	94.09
**10**	0.08	0.01	0.24	7.26	92.41
**11**	0.05	0.01	0.30	7.25	92.39
**12**	0.11	0.03	0.45	6.14	93.27
**13**	0.01	0.00	0.12	5.21	94.66
**14**	0.06	0.02	0.46	8.27	91.19
**15**	0.02	0.01	0.31	3.98	95.69
**Mean**	**0.05**	**0.01**	**0.30**	**6.39**	**93.24**
**Median**	**0.03**	**0.01**	**0.31**	**6.27**	**93.33**
**Max**	**0.13**	**0.03**	**0.46**	**10.25**	**97.01**
**Min**	**0.01**	**0.00**	**0.08**	**2.87**	**89.26**

Heavy metals in soils are present in different chemical forms with a wide variety of solubility or bioavailability. Partitioning of trace metals among various soil fractions in a given soil depends on the soil fractions, affinity to the metals, soil composition and fraction concentrations in the soils, metal concentration in soil solution, and soil pH etc. [36]. Researches on W chemical forms are extremely limited. Wilson and Pyatt [15] investigated W chemical forms in the acidic spoils in the vicinity of an abandoned W mine, showing about 99% of W in the RES fraction. However, in the calcareous soils in the vicinity of an ancient metalliferour mine in the Corbières Area (Southwestern France), W content ranged from 20.5 to 77.9 mg kg^−1^, of which about 41.2%, 23.0%, 19.1%, and 16.8% were bound in the exchangeable and carbonates, Fe/Mn oxides, soil organic matter, and residual fractions, respectively [17]. Therefore, W is more readily mobilized under alkaline conditions [17], [37]. In general, mobility and bioavailability of W might be relatively low in the acidic soils of Dayu county.

### Contents of W in the rice plants

The contents of W in the root, stem, leaf, and grain ranged from 1.57 to 25.76, 0.23 to 7.50, 0.59 to 16.66, and 0.02 to 0.57 mg kg^−1^, respectively (Table 3). The W content and its enrichment factor in rice follow the order: root > leaf > stem > grain. This order is similar to that for wild *Calluna Vulgaris*, *Taraxacum officinale*, and *Trifolium pretense* reported by Wilson and Pyatt [15] and Jiang et al. [18], i.e., W content in root > W content in leaf > W content in stem. In addition, content and enrichment in the root of *Sunflowers, Ryegrass(Lolium perenne), Nothofagus menziesii*, *wheat*, and *cowpea* growing in W-spiked soils were also higher than those in their leaves [2], [12], [20], [28], [29]. The common range of W in terrestrial plants was <0.001 to 0.15 mg kg^−1^ [6]. For example, the winter wheat grain, the spring barley grain, the wild berry grown in Sweden contained, in average, 0.006, 0.005, <0.01 mg kg^−1^ of W, respectively [23], [38]. The content of W in the Danish onion ranged from 0.006 to 0.039 mg kg^−1^, with an average of 0.017 mg kg^−1^ [22]. Twenty-two crop materials from Iowa contained 0 to 0.35 mg kg^−1^ of W [39]. However, plants grown in the W-contaminate soils usually contain high content of W. The maximum W levels of 50.7 mg kg^−1^ were found in cabbage, 41.2 mg kg^−1^ in onions and 12.3 mg kg^−1^ in potatoes grown in the W-contaminated soil adjacent to the W trioxide production plant [19].

**Table 3:** W contents in rice root, stem, leaf, and grain and its enrichment factor in them.

	W content				W enrichment factor			
Sample	Root	Stem	Leaf	Grain	Root	Stem	Leaf	Grain
	mg/kg	mg/kg	mg/kg	mg/kg				
**1**	2.34	0.70	0.85	0.12	0.285	0.086	0.104	0.015
**2**	25.76	7.50	16.66	0.57	0.590	0.172	0.382	0.013
**3**	7.09	1.64	9.64	0.33	0.437	0.101	0.594	0.020
**4**	10.27	4.34	5.33	0.36	0.367	0.155	0.190	0.013
**5**	4.64	1.42	3.71	0.08	0.369	0.113	0.295	0.006
**6**	5.83	1.49	1.83	0.08	0.342	0.087	0.107	0.005
**7**	4.26	2.34	4.13	0.09	0.280	0.154	0.272	0.006
**8**	5.44	2.21	3.78	0.09	0.351	0.143	0.244	0.006
**9**	21.75	6.30	14.41	0.40	0.573	0.166	0.380	0.010
**10**	2.30	1.45	2.58	0.07	0.221	0.139	0.248	0.006
**11**	1.57	1.12	2.57	0.05	0.297	0.212	0.488	0.010
**12**	1.90	0.72	1.45	0.04	0.478	0.181	0.364	0.010
**13**	2.23	0.23	0.59	0.02	0.524	0.055	0.138	0.005
**14**	2.24	0.59	1.75	0.07	0.449	0.118	0.351	0.014
**15**	8.31	3.10	2.19	0.18	0.328	0.122	0.086	0.007
**Mean**	**7.06**	**2.34**	**4.76**	**0.17**	**0.393**	**0.134**	**0.283**	**0.010**
**Median**	**4.64**	**1.49**	**2.58**	**0.09**	**0.367**	**0.139**	**0.272**	**0.010**
**Max**	**25.76**	**7.50**	**16.66**	**0.57**	**0.590**	**0.212**	**0.594**	**0.020**
**Min**	**1.57**	**0.23**	**0.59**	**0.02**	**0.221**	**0.055**	**0.086**	**0.005**

Rice is the main cereals food for the local residents in Dayu county. Assuming that local residents consume 0.4 kg rice per day per person, maximal dietary exposure to W for consumers of contaminated rice was estimated to be up to 0.23 mg W d^−1^ per person corresponding to the highest W content in rice grain. On the other hand, the exposure to W for the residents via soil ingestion was estimated to be 0.002 W mg d^−1^ per person corresponding to the highest W content in the soil, assuming that soil ingestion rate is 50 mg d^−1^ per person [40]. At an industrial production site for W trioxide in Switzerland, dietary exposure to W for consumers of contaminated vegetables (cabbage, onions, and potatoes) growing in garden was estimated to be up to 1.5 mg W d^−1^ per person compared to background dietary exposure of approximately 0.01 mg W d^−1^ per person [19]. Therefore, the major way of W exposure to the residents at W contaminated sites is the dietary ingestion. Although permissible exposure limit for W in workplace atmospheric environments in USA (5 mg m^−3^) and drinking water in the former USSR (0.05 mg L^−1^) were established, the threshold value for W content in any plant has not been established up to now from the viewpoint of food safety [2]. Tungsten health risk caused by the rice ingestion can not be quantitatively estimated at current stage.

The enrichment factors, defined as W contents in root, stem, leaf, and grain divided by its content in the soil, were 0.221 to 0.590 for root, 0.055 to 0.212 for stem, 0.086 to 0.594 for leaf, and 0.005 to 0.020 for grain. Previous studies showed the enrichment factor of W in the wild shrub shoots growing in the W mining areas (20.5–77.9 mg W kg^−1^ soil) was generally 0.01 to 0.8 [17]. The enrichment factors of W in the root and the leaf were 0.05–0.28 and 0.01–0.03, respectively, for *Taraxacum officinale* and *Trifolium pretense* growing in the fallowland soil (15.9 mg W kg^−1^ soil) down the hill from the Höllberg W mine in Germany [18]. Therefore, the W enrichment factor values in the rice root and leaf in this studied area were higher than those for the herb *Taraxacum officinale* and *Trifolium pretense*.

The contents of W in the root, stem, leaf, and grain of rice linearly increased with the content of W in the soil (Table 4), with the determination coefficients of 0.7178 to 0.9496. Several previous studies observed the dependence of plant W content on the soil W contents [3], [18], [28]–[30]. Previous experiments in field showed W contents in the roots and leaves of maize were significantly correlated with the W content in the soil adjacent to an old tungsten mine (Pechtelsgrün) in Germany [18]. In detail, W contents in the roots and leaves of maize increased from about 1 to 16 mg kg^−1^ and 0.4 to 1.5 mg kg^−1^, respectively, with the increase of soil W content from about 20 to 100 mg kg^−1^ [18]. In addition, W concentrations of roots and leaves both of wild *Taraxacum officinale and Trifolium pratense* were significantly correlated with soil W concentration. And there was a significant correlation between root and leaf tungsten concentrations for both species [18]. Pot experiment in greenhouse also demonstrated that W contents in root and shoot of sunflowers, ryegrass, wheat, and cowpea showed a very strong linear dependence on the W content in the W-spiked soils [3], [20], [28], [29] (Table 4).

**Table 4:** Relationship between the contents of W in plants (Y, mg W kg^−1^ dry weight) and in soils (X, mg kg^−1^) for W mining field investigations and pot experiments with W-spiked soils.

Plant	Soil W conc.	Duration	Equation	R^2^	n	Ref.
	mg/kg					
**Field investigation**						
Rice	3.99–43.65					This study
Root			Y = 0.5692X−2.3728	0.9141	15	
Stem			Y = 0.1703X−0.4797	0.9496	15	
Leaf			Y = 0.3404X−0.8781	0.7178	15	
Grain			Y = 0.0121X−0.0306	0.8161	15	
Taraxacum officinale	15.9–101.4					[18]
Root			Y = 0.1757X−1.229	0.8898	7	
Leaf			Y = 0.0132X+0.1979	0.8259	7	
Trifolium pratense	18.2–97.7					[18]
Root			Y = 0.1059X+2.5793	0.6139	7	
Leaf			Y = 0.0121X+0.1885	0.7048	7	
**Pot experiment with W-spiked soils**						
Nothofagus menziesii	0–125	8 weeks				[26]
Root			Y* = 32.205X+182.28	0.9778	12	
Stem (woody)			Y* = 0.7051X+19.313	0.7509	12	
Leaf			Y* = 0.9234X+9.8788	0.9009	12	
Sunflower	0–3900	28 days				[20]
Root			Y = 1.7098X+157.46	0.9863	5	
Leaf			Y = 0.0384X+16.234	0.9633	5	
Ryegrass	0–1000	9 months				[2]
Shoot			Y = 0.1916X+11.267	0.9935	5	
Wheat	0–120	up to fruiting				[28]
Root			Y = 0.88X−2.66	0.9716	6	
Shoot			Y = 17.50X−204.44	0.9288	6	
Cowpea	0–25	up to fruiting				[29]
Root			Y = 0.73X+6.3152	0.9661	6	
Shoot			Y = 0.23X+2.7685	0.9609	6	

*mg W kg^−1^ ash weight, R^2^: correlation coefficient, n: number of samples, Ref.: reference.

### Correlation among elemental contents, W chemical forms, and W contents in rice

The correlation matrix in Table 5 shows the relationships between elemental contents in soil, W chemical forms in soil, and W contents in rice. pH value was positively correlated to the contents of Mn, Sc, Al, Fe, W, W chemical forms, and W in rice, whereas these correlations are not significant at *p = 0.01* level. In general, the mineral matrix elements Mn, Sc, Al and Fe in the soil were correlated to one another, but they were not correlated to W content and chemical forms in the soil and the W contents in the rice. This might further inferred that the Mn, Sc, Al, and Fe contents in the soil mainly originated from geogenic sources, while W contents in the soil and rice were impacted by W mining activities. Tungsten contents in the soil and rice and W chemical forms in the soil were correlated to one another. These correlations showed that W mining activities in the Dayu county not only increased the total W content in soil, but also increased the W contents in each chemical form and in rice.

**Table 5:** Correlation matrix among mineral matrix element, W content and chemical forms in soil, and W contents in rice root, stem, leaf, and grain.

	pH	Mn	Sc	Al	Fe	TW	EXC-W	CARB-W	OX-W	OM-W	RES-W	R-W	S-W	L-W	G-W
pH	1.000	0.341	0.406	0.384	0.130	0.242	0.302	0.330	0.247	0.277	0.238	0.156	0.283	0.149	0.238
Mn		1.000	0.914*	0.933*	0.915*	0.237	−0.095	0.031	0.118	0.077	0.248	0.181	0.174	0.058	0.256
Sc			1.000	0.982*	0.903*	0.255	−0.024	0.118	0.143	0.063	0.269	0.211	0.185	0.014	0.234
Al				1.000	0.897*	0.284	−0.023	0.115	0.188	0.101	0.297	0.263	0.240	0.078	0.280
Fe					1.000	−0.002	−0.291	−0.161	−0.142	−0.205	0.014	−0.010	−0.083	−0.127	0.042
TW						1.000	0.708*	0.768*	0.969*	0.933*	1.000*	0.956*	0.975*	0.847*	0.903*
EXC-W							1.000	0.969*	0.807*	0.861*	0.692*	0.768*	0.773*	0.736*	0.680*
CARB-W								1.000	0.849*	0.869*	0.755*	0.811*	0.811*	0.742*	0.776*
OX-W									1.000	0.964*	0.964*	0.958*	0.990*	0.856*	0.897*
OM-W										1.000	0.922*	0.922*	0.951*	0.870*	0.871*
RES-W											1.000	0.953*	0.971*	0.841*	0.901*
R-W												1.000	0.968*	0.927*	0.916*
S-W													1.000	0.879*	0.899*
L-W														1.000	0.911*
G-W															1.000

Note: R-W: root W content, S-W: stem W content, L-W: leaf W content, G-W: grain W content. * Significant at *p = 0.01* level.

Plants are known to take up (possibly in anionic form, WO_4_^2−^) and accumulate W in substantial amounts [2], [25]. Whereas 89% to 97% of W in the studied soil was associated to the residual fraction, non-residual content of W was 0.23 to 3.58 mg kg^−1^, similar to W content (1.50 mg kg^−1^) in worldwide soils [35]. In addition, W content in the non-residual fractions was positively correlated to the total content of W in the studied soil. Therefore, the contents of W in rice were positively correlated to the total content of W in the soil. Previous studies also showed that the extent of W accumulation in plants appears to be directly related to the W content of the soils whereas chemical forms of W in the soils were not measured [2], [3], [18], [20], [26], [28], [29].

## Conclusions

About 100 years' mining W activity significantly elevated W contents in the soil adjacent to the W mines of the Dayu county. The rice grown in the soil accumulated W, with the linear dependence of W contents in the rice root, stem, leaf, and grain on the soil W content and chemical forms. The enrichment factor of W decreased in the order: root, leaf, stem, and grain. Most of W (about 93% in average) the soil was associated to the residual fraction. Tungsten contents and chemical forms in the soil and W contents in the rice root, stem, leaf, and grain were correlated to one another, but not correlated to the mineral matrix elemental (Mn, Sc, Al, and Fe) contents, due to the soil W contamination by W mining activities. The W exposure to local habitants via dietary and soil should be assessed further.
